# Evaluation of Cyfluthrin and Etofenprox Resistance in House Fly *Musca domestica* Populations in Antalya, Türkiye

**DOI:** 10.3390/biology13100767

**Published:** 2024-09-26

**Authors:** Emre Oz

**Affiliations:** Department of Medical Services and Techniques, Vocational School of Health Services, Antalya Bilim University, 07190 Antalya, Türkiye; emre.oz@antalya.edu.tr

**Keywords:** cyfluthrin, etofenprox, house fly, *Musca domestica*, resistance

## Abstract

**Simple Summary:**

House flies are important carriers of diseases and can cause economic losses in livestock by reducing feeding activity. Overuse of chemical insecticides has led to resistance in many house fly populations worldwide. This study aimed to assess how two house fly populations in Antalya, Türkiye respond to the insecticides cyfluthrin and etofenprox. By exposing adult flies to these chemicals, the researcher found that the Kemer population showed very low resistance to cyfluthrin, while the Serik population exhibited moderate resistance. For etofenprox, Kemer flies showed no resistance, and Serik flies showed very low resistance. The findings suggest that integrated pest control methods and regular resistance monitoring are necessary to manage and prevent further resistance in house fly populations.

**Abstract:**

The house fly, *Musca domestica* L. (Diptera: Muscidae), is a significant vector for many pathogens and parasites. Presence of this vector causes economic losses due to decreased feeding activity in livestock, resulting in reduced yields of products. The repeated and unconscious application of chemical insecticides has resulted in the development of resistance in the majority of house fly populations worldwide. The aim of this research was to determine the susceptibility levels of two field *M. domestica* populations to cyfluthrin and etofenprox in Antalya, Türkiye. The LD_50_ values, resistance ratios, and resistance status were determined by exposing adult house flies to the test chemicals for one hour. The Kemer population exhibited very low resistance to cyfluthrin, with a 5-fold increase, while the Serik population, in contrast, showed moderate resistance with a 29.67-fold increase. The resistance ratios of field populations to etofenprox were 2.33-fold (no resistance) for Kemer and 6.44-fold (very low resistance) for Serik, respectively. This is the first study to determine the resistance levels of house flies against cyfluthrin and etofenprox insecticides in Türkiye. To prevent or reduce the development of resistance to insecticides in house flies, integrated control methods and resistance management programs should be employed. Furthermore, it is advised that regular monitoring tests be conducted to observe the level of resistance.

## 1. Introduction

The house fly, *Musca domestica* L. (Diptera: Muscidae), is a cosmopolitan and synanthropic pest fly species that poses a significant health risk to humans and domestic animals. It is capable of thriving in a variety of environments, including barns, compost heaps, and garbage dumps. In particular, manure on animal farms provides an optimal habitat for the development of house flies. It is a carrier of over 200 pathogens, including bacteria, viruses, helminths, protozoa, fungi, and an arthropod, causing diseases such as hepatitis, cholera, dysentery, and tuberculosis in humans and livestock [1]. The transmission of organisms by flies occurs via their mouthparts, legs, feces, and vomit. A high density of house flies can have a detrimental impact on livestock farming, potentially leading to stress in the animals and a reduction in the economic value of their products [2,3].

Vector-targeted strategies are the primary means of managing house flies. However, as it is not sufficient to use only physical or biological methods to control this species, chemical control methods are also used in combination with these methods. For several decades, the management of house flies has been, and continues to be, dependent on the application of insecticides, including pyrethroids, carbamates, insect growth regulators, and organophosphates. The majority of insecticide formulations permitted by the Turkish Ministry of Health for use against house flies consist of synthetic pyrethroid, neonicotinoid, and insect growth regulator groups. Unfortunately, overuse and indiscriminate use of synthetic insecticides has led to the development of resistance in house flies against these chemicals in Türkiye and globally [4,5,6,7,8]. The issue of insecticide resistance represents a significant and growing challenge for the chemical control of insect pests. To overcome these negative situations, integrated pest management and resistance management programs should be employed against harmful insects, including limiting the use of insecticides, planned rotation, and/or the use of carefully selected mixtures.

House flies have evolved resistance to pyrethroids through two main mechanisms: voltage-sensitive sodium channel (Vssc) mutations and enhanced detoxification mediated by cytochrome P450 monooxygenases [9]. Etofenprox is a non-ester pyrethroid insecticide with a mode of action similar to that of other pyrethroids [10]. The active substance cyfluthrin belongs to the group of α-cyano pyrethroids. In Türkiye, these insecticides are used for the control of public health pests, including mosquitoes, cockroaches, and house flies. They have toxic effects on several insect species, including mosquitoes and planthoppers [10,11,12,13]. A review of the literature reveals a multitude of studies indicating that house flies have developed high levels of resistance to a range of insecticide groups globally, including in Türkiye [14,15,16,17]. However, no study has been found to have been conducted with cyfluthrin and etofenprox in Türkiye. In this study, we investigated the resistance levels of house fly populations collected from animal barns in the Kemer and Serik districts of Antalya province in Türkiye against the active ingredients cyfluthrin and etofenprox.

## 2. Materials and Methods

### 2.1. Test Chemicals

Etofenprox (C_2_₅H_28_O_3_, CAS number: 80844-07-1, molecular weight: 376.49 g/mol) and cyfluthrin (C_22_H_18_Cl_2_NO_3_, CAS number: 68359-37-5, molecular weight: 434.29 g/mol), purchased from Sigma-Aldrich (Darmstadt, Germany), were dissolved in acetone for use in tests. Following preparation of the stock solutions, dilutions were executed, and a minimum of six doses were tested in the study.

### 2.2. House Flies

House flies were collected using traps from barns, which are natural development and breeding areas, in the Kemer and Serik districts of Antalya, Türkiye (Table 1) in July 2023. The house flies, brought into the laboratory in tulle cages, were transferred again to clean fine muslin tulle cages (22 × 22 × 22 cm) and cultured. To promote the optimal development of individuals, a sufficient quantity of food items, including bran, milk, sugar, and water, was supplied. Individuals were reared in the Vector Ecology and Control laboratory at Akdeniz University under conditions of 25 ± 1 °C temperature, 60 ± 5% humidity, and a photoperiod of 12 h light and 12 h darkness. The insecticide-susceptible house fly population, obtained from Pavia University in Italy by Dr. Oner Kocak (from Hacettepe University, Pesticide Testing Laboratories) in 2004, has been reared under the same conditions for twenty years at the Vector Ecology and Control laboratory at Akdeniz University.

### 2.3. Resistance Tests

Resistance tests were carried out with reference to the standard surface method recommended by Polat and Cetin [8]. To ascertain the lethal dose fifty (LD_50_) value, a minimum of six doses were investigated, comprising both the World Health Organization (WHO)-recommended doses and upper and lower doses. The experiments were carried out in glass jars with a surface area of 260 cm^2^. The chemicals were dispersed on the inner surface of the jars using acetone as a solvent, and the jars were utilized in the experiments following a 24 h waiting period for the acetone to evaporate. A minimum of twenty-five adult house flies (mixed gender) aged 2–4 days were released inside the jars, and they were exposed to the active substance for a duration of one hour. After the exposure time ended, all individuals were transferred to clean jars, and cotton soaked with a 10% sugar solution was placed inside the glass jars. After 24 h, both alive and dead individuals were recorded. The control group was treated with acetone only on the inner surface of the jars, and the subsequent procedures were identical to those previously described. An individual was categorized as dead if it did not exhibit any movement or flight behavior at all. For each dose, a minimum of three replicates were conducted. All experiments were performed in a laboratory setting with a temperature of 26 ± 2 °C, humidity of 60 ± 5%, and a photoperiod of 12 h light and 12 h darkness.

### 2.4. Statistical Analysis

In experiments with mortality rates in the control group in the range of 5–20%, treatment mortality rates were corrected using the Abbott [18] formula. If the mortality rate in the control group exceeded 20%, the experiments were terminated and repeated to ensure the reliability of the results. All statistical analyses were conducted using the SPSS 20.0 software program (IBM Corp., Armonk, NY, USA). The data have been subjected to Kolmogorov–Smirnov and Shapiro–Wilk normality tests, and all data showed a normal distribution. Therefore, mean mortality data, dependent on dose and population, were subjected to analysis of variance (ANOVA). Significant differences between means were identified by a Tukey HSD test (*p* ≤ 0.05). The LD_50_ values of the populations were calculated after 24 h of exposure using probit analysis with the SPSS 20.0 software program [19]. The resistance level was determined by dividing the LD_50_ value obtained from the field by the LD_50_ value obtained from the susceptible population. The resistance levels were evaluated in five categories. According to these categories, the following resistance levels were identified: resistance ratio (RR) < 5 no resistance, RR = 5–10 very low resistance, RR = 11–20 low resistance, RR = 21–50 moderate resistance, RR = 51–100 high resistance, and RR > 100 very high resistance [20].

## 3. Results

Table 2 and Table 3 present a comparative analysis of the toxicological profiles of cyfluthrin and etofenprox. In all populations, mortality rates exhibited a general increase as the dose of the administered chemical increased, with a statistical difference observed between doses. The 0.03 g ai/m^2^ dose of cyfluthrin, which is recommended by the WHO for the control of houseflies, demonstrated significantly higher mortality (80.58%) in the Kemer population and a low mortal effect in the Serik population (32.33%). A statistical difference was observed between the populations at the 0.03 and 0.045 doses of cyfluthrin (Table 2). The Serik population showed a statistical difference compared to the WHO and Kemer populations at the 0.05 and 0.01 doses of etofenprox (Table 3). The dose of 0.1 g ai/m^2^, which is the dose recommended by the WHO for etofenprox, resulted in complete mortality in the Kemer population, while it caused a lower mortality rate (78%) in the Serik population.

Table 4 presents LD_50_ values, confidence limits, resistance ratios, and resistance statuses of cyfluthrin and etofenprox. The LD_50_ value of cyfluthrin for the susceptible population was calculated to be 0.003 g ai/m^2^. The Kemer and Serik populations exhibited LD_50_ values of 0.015 and 0.089 g ai/m^2^, respectively. The Kemer population exhibited very low resistance to cyfluthrin, with a five-fold increase. The Serik population, on the other hand, showed moderate resistance with a 29.67-fold increase.

The LD_50_ values of etofenprox for the WHO, Kemer, and Serik populations were calculated to be 0.009, 0.021, and 0.089 g ai/m^2^, respectively. The resistance ratios of field populations for etofenprox were found to be 2.33-fold and 6.44-fold for Kemer and Serik populations, respectively. Upon evaluating the resistance status, the Kemer population was found to have no resistance, while the Serik population had very low resistance. The resistance ratio was lower in the Kemer population than in the Serik population for both active ingredients.

## 4. Discussion

*Musca domestica* is a significant vector for the transmission of pathogens, including bacteria, viruses, and fungi, affecting humans and animals. To successfully combat house flies, it is necessary to understand their biology, ecology, and behavioral characteristics well. Synthetic pyrethroids and neonicotinoids have been employed in Türkiye for the control of adult house flies, while insect growth regulators are utilized for the control of larvae. Evaluating pest resistance to insecticides is crucial in selecting the most effective compound for pest management. Previous studies have documented the resistance status of the house fly to insecticides on poultry farms and solid waste landfills in different regions of Türkiye. Memmi et al. [6] reported that house fly populations collected from Antalya, Ankara, and İzmir provinces exhibited varying degrees of resistance to imidacloprid and methomyl insecticides. In a study conducted by Şişli et al. [21], the efficacy of three insecticides, namely malathion, fenitrothion, and propoxur, was evaluated on house flies collected from the Ankara Municipal dumpsite. The results indicated that the highest resistance was observed in malathion. In a study conducted by Eligül et al. [22], deltamethrin was tested on house fly populations collected from landfills, wastewater treatment plants, sugar factories, and city centers in Konya. The results showed that low mortality rates of ≤35.2% were found in the populations. Nevertheless, this is the first study, to our knowledge, to investigate the efficacy of cyfluthrin and etofenprox for house fly control in Türkiye.

The low RR value (five-fold) observed in the Kemer population in response to cyfluthrin in our study highlights a high level of susceptibility to the insecticide. In contrast, the Serik population demonstrated a more moderate level of resistance, with a 29.67-fold resistance. The low to moderate resistance of *M. domestica* to the insecticides used in the study may be attributed to the limited use of these active ingredients in Antalya. The results demonstrate the significance of the relationship between selection pressure and resistance management. Neonicotinoids and synthetic pyrethroids, especially alpha-cypermethrin, deltamethrin, and permethrin, are the most intensively used insecticides against *M. domestica* in the Antalya region. Numerous studies have demonstrated that house flies have developed high levels of resistance to neonicotinoid and synthetic pyrethroid insecticides. Erdogan and Çetin [15] investigated the level of deltamethrin resistance in five house fly populations from the Kumluca district in Antalya, Türkiye. The results indicated that moderate and high resistance levels were present in this region. In a study conducted by Koç et al. [23], flies collected from solid waste landfills in the Varsak district of Kepez in Antalya province exhibited medium-high levels of resistance to synthetic pyrethroids, including cypermethrin, cyphenothrin, deltamethrin, and permethrin. In a separate study conducted in Antalya province, extremely high resistance (≥114,000-fold) to thiamethoxam, which is a neonicotinoid insecticide, was observed in different regions. When the resistance levels were compared, a significant increase in resistance coefficients was recorded in regions with high animal production [16]. The higher resistance ratio observed in the Serik district is attributed to the higher pesticide application in this region in connection with the prevalence of greenhouse agriculture.

A number of studies conducted worldwide have demonstrated the resistance of *M. domestica* to cyfluthrin. Abbas and Hafez [24] reported that an alphacypermethrin-selected *M. domestica* population revealed moderate cross-resistance to cyfluthrin (16.8-fold). Hafez [24] conducted a study to test the effectiveness of eight commonly used insecticides, including cyfluthrin, against five populations of house flies collected from dairies around Riyadh, Saudi Arabia. The study found that the flies exhibited a one- to nine-fold resistance to cyfluthrin. Ong et al. [25] investigated the effect of cyfluthrin on *M. domestica* through topical application and plywood treatment. They reported that cyfluthrin has low LC_50_ values and resistance ratios of 1.26 and 3.29, respectively. Kaufman et al. [26] conducted a study on the resistance of seven insecticides, including cyfluthrin, against *M. domestica*. The highest levels of resistance were found for tetrachlorvinphos, permethrin, and cyfluthrin. Scott et al. [14] investigated the resistance of eight populations of house flies to nine insecticides (dimethoate, tetrachlorvinphos, permethrin, cyfluthrin, pyrethrins, methomyl, fipronil, spinosad, and cyromazine) collected from caged-layer poultry facilities across New York State. The study found that the highest levels of resistance were noted for tetrachlorvinphos, permethrin, and cyfluthrin. Pospischil et al. [27] reported that *M. domestica* isolated from farms in three different German districts had very high resistance to cyfluthrin, between 67.7 and 292-fold. Abu Nada and Nazer [28] found slight resistance to cyfluthrin with RFs of 10.84 against a field house fly population.

To date, no study has been conducted to assess the resistance of etofenprox to *M. domestica* worldwide. Nevertheless, the toxicity of etofenprox against a range of vectors, including house flies, cockroaches, and mosquitoes, has only been the subject of a limited number of studies [29,30]. Our study represents the first investigation into the resistance status of etofenprox against *M. domestica*. Upon evaluating the resistance status, the Kemer population was found to have no resistance (2.33-fold), while the Serik population had very low resistance (6.44-fold). The low or absent resistance of field populations to etofenprox can be attributed to the fact that this active substance is not used much in Antalya province. As previously stated, the most intensively employed insecticides for the control of adult house flies are alpha-cypermethrin, deltamethrin, permethrin, lambda-cyhalothrin, and thiamethoxam.

In the present study, the low level of resistance to pyrethroids in the populations suggests that these insecticides are still effective in controlling house flies in barns in Antalya, Türkiye. Nevertheless, it is of particular importance to monitor the Serik population for the development of resistance, given that it displays moderate resistance to cyfluthrin. The vector control studies conducted by the Metropolitan Municipality in Antalya have observed a significant increase in the utilization of pesticides in areas such as garbage containers, animal farms, waste storage, and separation facilities [31,32]. Inappropriate and excessive use of this insecticide could lead to the development of resistance in the future. Furthermore, this situation may give rise to cross- and multiple-resistance. Numerous studies have demonstrated that repeated exposure of an organism to the same insecticide can lead to increased resistance of that organism to insecticides. One study examined the evolution of resistance to alpha-cypermethrin selection (Alpha-Sel) in the house fly over 24 generations. It was observed that the resistance of females to alpha-cypermethrin increased from 46.4-fold to 474.2-fold, while that of males increased from 41.0-fold to 253.2-fold, in comparison to an alpha-cypermethrin-unselected population (Alpha-Unsel). Furthermore, while the Alpha-Sel population exhibited low cross-resistance (CR) to two additional pyrethroids and five organophosphates, moderate CR was observed in response to bifenthrin (15.5-fold), deltamethrin (28.4-fold), and cyfluthrin (16.8-fold) [33]. In another study, Abbas et al. [34] demonstrated the mode of resistance development to lambda-cyhalothrin in a laboratory. Their findings indicated that after 11 generations of selection against the susceptible population, resistance had increased by 113.57-fold.

Our results, along with studies conducted in other countries, have demonstrated that house flies have developed resistance to many insecticides. To prevent or reduce this resistance, an integrated pest management program should be implemented against house flies. Principal strategies for controlling resistance in house flies include the alternation of insecticides with different modes of action, the application of insecticides in more limited areas instead of large areas, the preparation of resistance maps, use of sticky traps, proper disposal of manure or other decaying organic matter to prevent pest breeding, and the use of synergistic substances such as PBO, DEF, and DEM based on the results of enzyme activities. Several studies conducted in Türkiye have demonstrated that vectors can be effectively controlled by the use of products containing a synergistic active ingredient, particularly in populations that are resistant to insecticides [8,35]. In addition, larvicides containing insect growth regulators, in particular, should be used on resistant house flies, as little or no cross-resistance to other insecticides has been identified, reducing insecticide-induced environmental damage [36].

## 5. Conclusions

Etofenprox and cyfluthrin have been shown to be effective in controlling house fly populations in barns in Antalya, Türkiye. However, it is important to closely monitor the Serik population due to its moderate resistance to cyfluthrin, indicating a potential for further resistance development. This study represents the first investigation into the resistance levels of house flies against cyfluthrin and etofenprox in Türkiye. To mitigate or prevent the escalation of insecticide resistance in house flies, it is essential to implement integrated pest management strategies and resistance management programs.

## Figures and Tables

**Table 1:** Global Positioning System coordinates, sample date, locality, and habitat types from which *Musca domestica* was sampled in Antalya, Türkiye.

Locality	Area Type	Sampling Date	Coordinates
Kemer	Barn	July 2023	N 36°34′13.2″ E 30°33′57.6″
Serik	Barn	July 2023	N 36°55′22.3″ E 31°07′52.6″

**Table 2:** The comparative toxicity (% mean mortality ± standard error) of various doses of cyfluthrin on *Musca domestica* populations.

Populations	Doses (g ai/m^2^)						
WHO	**0.00**	**0.0003**	**0.003**	**0.03**	**0.045**	**0.45**	
	4.95 ± 1.56 a,A	23.63 ± 1.47 a	65.82 ± 13.16 b	100 c,B	96.96 ± 1.52 c,B	100 c,A	
Kemer	**0.00**	**0.0015**	**0.002**	**0.03**	**0.045**	**0.45**	
	1.33 ± 1.33 a,A	12.81 ± 5.23 a	59.95 ± 13.98 b	80.58 ± 3.99 bc,B	91.36 ± 6.66 bc,B	100 c,A	
Serik	**0.00**		**0.002**	**0.03**	**0.045**	**0.** **45**	**0.** **3**
	10.16 ± 5.2 a,A		21.53 ± 8.62 ab	32.33 ± 12.31 ab,A	48.63 ± 2.83 b,A	93.06 ± 6.94 c,A	100 c

If lowercase letters are different on the same line, there is a statistical difference between doses, (Tukey HSD test, *p* ≤ 0.05). If capital letters are different in the same column, there is a statistical difference between doses, (Tukey HSD test, *p* ≤ 0.05).

**Table 3:** The comparative toxicity (% mean mortality ± standard error) of various doses of etofenprox on *Musca domestica* populations.

Populations	Doses (g ai/m^2^)						
WHO	**0.00**	**0.001**	**0.005**	**0.01**	**0.05**	**0.1**	**1**
	4.95 ± 1.56 a,A	10.07 ± 0.58 a,A	41.55 ± 6.66 b,A	81.97 ± 11.46 c,B	95.74 ± 2.64 c,B	100 c,B	100 c,A
Kemer	**0.00**	**0.001**	**0.005**	**0.01**	**0.05**	**0.1**	**1**
	8.00 ± 2.31 a,A	9.09 a,A	34.64 b,A	49.4 ± 9.11 b,AB	86.99 ± 16.55 c,B	100 c,B	100 c,A
Serik	**0.00**	**0.001**	**0.005**	**0.01**	**0.05**	**0.1**	**1**
	4.95 ± 1.56 a,A	14.10 ± 4.51 ab,A	25.14 ± 3.8 ab,A	26.88 ± 6.03 b,A	35.75 ± 5.44 b,A	78.57 ± 6.27 c,A	100 c,A

If lowercase letters are different on the same line, there is a statistical difference between doses, (Tukey HSD test, *p* ≤ 0.05). If capital letters are different in the same column, there is a statistical difference between doses, (Tukey HSD test, *p* ≤ 0.05).

**Table 4:** Lethal dose 50 values, confidence limits, Chi-square, degree of freedom (df), significance (*p*), resistance ratio, and resistance status against etofenprox and cyfluthrin in *Musca domestica* populations.

Cyfluthrin							
Populations	LD_50_ (g ai/m^2^)	Confidence Limits (95%)	Chi-Square ^a^	df	*p*	Resistance Ratio (RR)	Resistance Status
WHO	0.003 A	0–0.01	137.754	10	0.0001	1	
Kemer	0.015 B	0.005–0.025	340.070	16	0.0001	5	VERY LOW
Serik	0.089 C	0–0.18	382.981	13	0.0001	29.67	MODERATE
**Etofenprox**							
WHO	0.009 A	0–0.021	288.069	13	0.0001	1	
Kemer	0.021 B	0.012–0.031	140.792	16	0.0001	2.33	NO
Serik	0.058 C	0.047–0.072	70.231	16	0.0001	6.44	VERY LOW

^a^ Chi-square values are significant at *p* < 0.05 levels. If capital letters are different in the same column, there is a statistical difference between doses, (Tukey HSD test, *p* ≤ 0.05).

## Data Availability

Data are contained within the article.

## References

[1] Nayduch D., Burrus R.G. (2017). Flourishing in filth: House fly–microbe interactions across life history. Ann. Entomol. Soc. Am..

[2] Learmount J., Chapman P., Macnicoll A. (2002). Impact of an insecticide resistance strategy for house fly (Diptera: Muscidae) control in intensive animal units in the United Kingdom. J. Econ. Entomol..

[3] Förster M., Klimpel S., Sievert K. (2009). The house fly (*Musca domestica*) as a potential vector of metazoan parasites caught in a pig-pen in Germany. Vet. Parasitol..

[4] Liu N., Yue X. (2000). Insecticide resistance and cross-resistance in the house fly (Diptera: Muscidae). J. Econ. Entomol..

[5] Acevedo G.R., Zapater M., Toloza A.C. (2009). Insecticide resistance of house fly, *Musca domestica* (L.) from Argentina. Parasitol. Res..

[6] Memmi B.K. (2010). Mortality and knockdown effects of imidacloprid and methomyl in house fly (*Musca domestica* L., Diptera: Muscidae) populations. J. Vector Ecol..

[7] Cetin H., Kocak O., Oz E., Koc S., Polat Y., Arikan K. (2019). Evaluation of some synthetic pyrethroids and piperonyl butoxide combinations against Turkish house fly (*Musca domestica* L.) populations. Pak. J. Zool..

[8] Polat B., Çetin H. (2020). Toxicity of thiamethoxam and piperonyl butoxide combination against some strains of house fly *Musca domestica* L. (Diptera) in Turkey. Acta Zool. Bulg..

[9] Scott J.G. (2017). Evolution of resistance to pyrethroid insecticides in *Musca domestica*. Pest Manag. Sci..

[10] Hemingway J. (1995). Efficacy of etofenprox against insecticide susceptible and resistant mosquito strains containing characterized resistance mechanisms. Med. Vet. Entomol..

[11] Fonseca-González I., Quiñones M.L., Lenhart A., Brogdon W.G. (2011). Insecticide resistance status of *Aedes aegypti* (L.) from Colombia. Pest Manag. Sci..

[12] Sun H., Yang B., Zhang Y., Liu Z. (2017). Metabolic resistance in *Nilaparvata lugens* to etofenprox, a non-ester pyrethroid insecticide. Pestic. Biochem. Physiol..

[13] Li Y., Zhou G., Zhong D., Wang X., Hemming-Schroeder E., David R.E., Lee M.-C., Zhong S., Yi G., Liu Z. (2021). Widespread multiple insecticide resistance in the major dengue vector *Aedes albopictus* in Hainan Province, China. Pest Manag. Sci..

[14] Scott J.G., Alefantis T.G., Kaufman P.E., Rutz D.A. (2000). Insecticide resistance in house flies from caged-layer poultry facilities. Pest Manag. Sci..

[15] Erdogan G., Cetin H. (2020). Survey of deltamethrin resistance in house flies (*Musca domestica* L.) collected from Kumluca which is the most important greenhouse production area of Turkey. Fresenius Environ. Bull..

[16] Çakır D., Çetin H. (2021). Determination of resistance levels against thiamethoxam in house fly (*Musca domestica* L.) populations in Antalya. Turk. Parazitoloji Derg..

[17] Pai H.H., Chang C.Y., Lin K.C., Hsu E.L. (2023). Rapid insecticide resistance bioassays for three major urban insects in Taiwan. Parasites Vectors.

[18] Abbott W.S. (1925). A method of computing the effectiveness of an insecticide. J. Econ. Entomol..

[19] Finney D.J. (1971). Probit Analysis.

[20] Wang J.N., Hou J., Wu Y.Y., Guo S., Liu Q.M., Li T.Q., Gong Z.Y. (2019). Resistance of house fly, *Musca domestica* L. (Diptera: Muscidae), to five insecticides in Zhejiang province, China: The situation in 2017. Can. J. Infect. Dis. Med. Microbiol..

[21] Şişli M.N., Boşgelmez A., Koçak O., Porsuk H. (1983). The effect of malathion, fenitrothion and propoxur on the house fly, *Musca domestica* L. (Diptera: Muscidae) populations. Microbiol. Bull..

[22] Eligül H., Koçak Ö., Çökmüş C. Konya ili ev sineği (*Musca domestica* L.) popülasyonlarında deltamethrin’in etkisinin incelenmesi. Proceedings of the III Ulusal Vektör Mücadelesi Sempozyumu.

[23] Koç S., Oz E., Erdogan G., Yanikoglu A., Cetin H. (2012). Synthetic pyrethroid resistance in house fly, *Musca domestica* L. (Diptera: Muscidae), from the solid waste collection facility of Varsak, Antalya, Turkey. Fresenius Environ. Bull..

[24] Hafez A.M. (2021). First evaluation of field evolved resistance to commonly used insecticides in house fly populations from Saudi Arabian dairy farms. Insects.

[25] Ong S.Q., Ahmad H., Jaal Z., Rus A.C. (2016). Comparative effectiveness of insecticides for use against the house fly (Diptera: Muscidae): Determination of resistance levels on a Malaysian poultry farm. J. Econ. Entomol..

[26] Kaufman P.E., Scott J.G., Rutz D.A. (2001). Monitoring insecticide resistance in house flies (Diptera: Muscidae) from New York dairies. Pest Manag. Sci..

[27] Pospischil R., Szomm K., Londershausen M., Schröder I., Turberg A., Fuchs R. (1996). Multiple resistance in the larger house fly *Musca domestica* in Germany. Pestic. Sci..

[28] Abu Nada Y.A., Nazer I.K. (1996). Response of the house fly *Musca domestica* L.(Diptera: Muscidae) in the central Jordan Valley to eight insecticides. Arab. Gulf J. Sci. Res..

[29] Putsintseva L.S., Dremova V.P., Labzin V.V., Gitsu F.V., Dem’yanov E.V. (1992). Efficacy of the new insecticide ethofenprox (Trebon) in the control of different species of insects. Med. Parazitol..

[30] Putintseva L.S., Dremova V.P. (1992). Comparative evaluation of the insecticidal activity of pyrethroid-based preparations. Med. Parazitol..

[31] Çetin H. (2002). Antalya Kenti Sivrisinek (Diptera: Culicidae) Türleri, Yaşama Alanları ve Savaşımlarına Ilişkin bir Araştırma. Master’s Thesis.

[32] Çetin H. (2018). Belediyeler açısından vektör (haşere) mücadelesinin temel sorunları. Turk. Belediyeler Birl. Derg..

[33] Abbas N., Hafez A.M. (2023). Alpha-cypermethrin resistance in *Musca domestica*: Resistance instability, realized heritability, risk assessment, and insecticide cross-resistance. Insects.

[34] Abbas N., Khan H.A.A., Shad S.A. (2014). Resistance of the house fly *Musca domestica* (Diptera: Muscidae) to lambda-cyhalothrin: Mode of inheritance, realized heritability, and cross-resistance to other insecticides. Ecotoxicology.

[35] Oz E., Cetin H. (2022). Synergistic effect of piperonyl butoxide on the toxicity of alpha-cypermethrin and deltamethrin against pyrethroid-resistant german cockroach *Blattella germanica* (Blattodea: Ectobiidae) strains in Turkey. Int. J. Trop. Insect Sci..

[36] Hafez A.M. (2022). Risk assessment of resistance to diflubenzuron in *Musca domestica*: Realized heritability and cross-resistance to fourteen insecticides from different classes. PLoS ONE.

